# Prevalence of Gastroesophageal Reflux Symptoms Post Sleeve Gastrectomy in Al-Qassim Region

**DOI:** 10.7759/cureus.44040

**Published:** 2023-08-24

**Authors:** Khaled A Alnafisah, Faisal A Alamer, Noura I Alotayk, Renad Khalid, Haifa N Alsaleem, Thekra Bennasser, Maha Alsaif, Faisal T Alayed, Ammar M Al Ammari

**Affiliations:** 1 Department of Gastroenterology, King Fahad Specialist Hospital, Buraydah, SAU; 2 College of Medicine, Qassim University, Buraydah, SAU

**Keywords:** quality of life, regurgitation, heartburn, sleeve gastrectomy, gastroesophageal reflux symptoms

## Abstract

Background and aims: After sleeve gastrectomy, heartburn sensation and regurgitation are frequent postoperative consequences. This study aimed to determine the prevalence and severity of heartburn sensation and regurgitation symptoms among patients who underwent sleeve gastrectomy, as well as the relationship between demographic variables and the presence and severity of these symptoms.

Methodology: This cross-sectional study included 290 patients who underwent sleeve gastrectomy in the Al-Qassim region, Saudi Arabia. Patients were asked to complete a questionnaire that assessed the presence and severity of heartburn and regurgitation symptoms. Demographic data, including age, gender, smoking status, and the date of bariatric surgery, were also collected.

Results: The results showed that heartburn and regurgitation were common symptoms among patients who underwent sleeve gastrectomy, with 78% and 73.9% of patients reporting these symptoms, respectively. 11.5% of patients who experienced severe symptoms of regurgitation, and 6.4% of patients with severe heartburn reported serious symptoms that affected their lives by causing an inability to perform daily activities. Age and the date of bariatric surgery were significant factors associated with heartburn and regurgitation symptoms. Patients aged 25-35 years reported the highest prevalence of heartburn symptoms, and the more recent surgery; dated less than one year ago had the highest prevalence of heartburn symptoms.

Conclusion: Patients who have sleeve gastrectomy frequently experience heartburn and regurgitation, which can have a substantial influence on their quality of life. Routine evaluation and treatment of heartburn and regurgitation symptoms should be an integral component of postoperative care. Younger patients and those who undergo surgery in the early postoperative period may be at a greater risk for developing heartburn and regurgitation symptoms, necessitating more active measures to treat these symptoms.

## Introduction

Obesity is a global healthcare threat affecting both adults and children and nowadays it presents a high-cost nutritional problem [1]. Obesity is identified as fat building up abnormally, a BMI of greater than 30 kg/m^2^ is interpreted as obesity according to the World Health Organization, it is associated with several health issues such as diabetes mellitus, cardiovascular diseases, and particular types of cancer (breast, colon and endometrial) [2,3]. It is estimated that more than 300 million adults worldwide are obese and 20% are morbidly obese (BMI>35 kg/m^2^) [4].

Bariatric surgery aids in the reduction of mortality and morbidity rates in obese patients, one of the most widely performed bariatric surgeries worldwide is sleeve gastrectomy (SG) because of its multiple advantages among which are the short operative time, low risk of post-operative complications, the lack of foreign material, the lack of gastrointestinal anastomosis, and malabsorption [1,5].

Nevertheless, gastroesophageal reflux disease (GERD) is a significant side-effect following SG [4]. It develops when the stomach content regorges to the esophagus and causes heartburn and irritation, negatively impacting the patient’s life [6]. The association between GERD and SG is still unclear but could be explained by the increase in intragastric pressure due to alterations of stomach shape into a long narrow tube resulting in a decrease in gastric compliance and by disturbing the anatomical reflux mechanism [1].

There is insufficient but statistically significant evidence regarding the higher occurrence of de-novo GERD, especially in elderly and co-morbid patients compared to healthy individuals. In a study done in Saudi Arabia, they found that 14.6% of patients who underwent SG were diagnosed with GERD, though 11.5% of them were diagnosed before SG, demonstrating a significant clinical association between the time of LSG and GERD [1,7].

It is important to determine the prevalence of GERD after SG, as well as the risk factors for it, and its impact on quality of life. In addition, the literature shows that there has been no research in the Al-Qassim area to determine the prevalence and risk factors for GERD after SG. Thus, we aim to investigate this area of research and implement this study to increase the identification of impacted cases and implement the proper management plan.

## Materials and methods

A cross-sectional descriptive study was conducted in the Al-Qassim region, Saudi Arabia, from March 2023 to December 2023. The sample size for this study was 290 patients who had undergone SG in the Al-Qassim region. Participants were selected using a convenience sampling technique, with inclusion criteria being adult patients (>18y) who had done LSG in the Al-Qassim region, and exclusion criteria being pediatric patients (<18y), patients who had pre-LSG endoscopy and were diagnosed as Barrett's esophagus, patients who had complications after surgery, and patients who did not complete the GERD-HRQL questionnaire.

Data collection was performed using an online survey on social media apps such as WhatsApp, Twitter, etc. The survey included the GERD-HRQL questionnaire and questions related to patient demographics, pre and post-operative weight, BMI, and severity of GERD symptoms. The variables that were collected in this study included age, gender, nationality, height, pre-operative weight, post-operative weight, pre-operative BMI, post-operative BMI, the severity of heartburn, the severity of heartburn when lying down, severity of heartburn when standing, the severity of heartburn after meals, heartburn requiring a change in diet, heartburn disturbing sleep, the severity of dysphagia, the severity of odynophagia, regurgitation of heartburn, regurgitation when lying down, regurgitation when standing, regurgitation after meals, regurgitation requiring change in diet, regurgitation disturbing sleep, medication affecting daily life, and general satisfaction.

Data analysis was performed using Statistical Package for the Social Sciences (SPSS; IBM Corp., Armonk, NY), with collected data being coded and presented using simple percentages. A probability level (p-value) of 0.05 or less was used to indicate statistical significance.

To ensure ethical considerations were met, ethical approval was sought from the Al-Qassim Research Ethics Committee (QREC) prior to the commencement of the study. The survey data was kept entirely confidential, and only researchers were able to access the survey's data. Informed consent was obtained from all participants before participating in the study.

## Results

Table 1 shows the demographic factors of the patients who underwent SG in Al-Qassim region, Saudi Arabia. The study included 290 patients, with 169 (57.3%) males and 126 (42.7%) females. The majority of the patients were between the ages of 25-35 (36.3%), followed by those who were less than 25 years old (25.4%). Only 29.5% of the patients reported being smokers. The date of the bariatric surgery varied among patients, with 20.7% having undergone the procedure less than a year ago, and 27.8% having done it more than five years ago. The mean weight before surgery was 122.89 kg (SD=28.98), while the mean weight after surgery (current weight) was 73.83 kg (SD=14.77). The mean height of the patients was 163.85 cm (SD=18.94). In addition, it was found that 55.3% of the participants reported using reflex medications.

**Table 1:** Demographic factors of the patients

		Count	Column, N%
Gender	Male	169	57.3
	Female	126	42.7
Age	Less than 25	75	25.4
	25-35	107	36.3
	36-45	66	22.4
	> 45	47	15.9
Are you smoker?	No	208	70.5
	Yes	87	29.5
The date of the bariatric surgery	Less than one year	61	20.7
	1-2 years	72	24.4
	3 years	35	11.9
	4 years	26	8.8
	5 years	19	6.4
	> 5 years	82	27.8
Weight before the bariatric surgery	Mean (SD)	122.89	28.98
Weight after the bariatric surgery (current weight)	Mean (SD)	73.83	14.77
Height (cm)	Mean (SD)	163.85	18.94
Do you take any reflex medication?	No	132	44.7
	Yes	163	55.3

Table 2 shows the prevalence of heartburn symptoms among the participants who underwent SG in Al-Qassim region, Saudi Arabia. The study included 290 patients, and the prevalence of heartburn symptoms was assessed using the GERD-HRQL questionnaire. The results show that 22.0% of the patients reported no symptoms of heartburn, while 6.4% reported an inability to perform daily activities due to heartburn. Heartburn when lying down was reported by 26.1% of the patients, and heartburn when standing up was reported by 42.7% of the patients. In terms of heartburn after meals, 24.1% of the patients reported noticeable symptoms, but not bothersome, while 12.5% reported bothersome symptoms daily. Changes in diet due to heartburn were reported by 36.6% of the patients. 41.7% of the patients reported that heartburn woke them from sleep. Difficulty swallowing was reported by 52.9% of the patients, while 58.3% reported pain while swallowing. The severity of regurgitation was classified as bothersome and affecting daily activities by 9.5% of the patients, while 11.5% reported an inability to perform daily activities due to regurgitation.

**Table 2:** The prevalence of heartburn symptoms among the participants

	No symptoms		Noticeable , but not bothersome		Noticeable, bothersome but not every day		Bothersome daily		Bothersome and affects daily activities		Inability to perform daily activities	
	Count	Row, N%	Count	Row, N%	Count	Row, N%	Count	Row, N%	Count	Row, N%	Count	Row, N%
How bad is the heartburn?	65	22.0	77	26.1	72	24.4	42	14.2	20	6.8	19	6.4
Heartburn when lying down?	77	26.1	64	21.7	69	23.4	39	13.2	16	5.4	30	10.2
Heartburn when standing up?	126	42.7	73	24.7	52	17.6	29	9.8	10	3.4	5	1.7
Heartburn after meals?	69	23.4	62	21.0	71	24.1	37	12.5	24	8.1	32	10.8
Does heartburn change your diet?	108	36.6	51	17.3	53	18.0	37	12.5	16	5.4	30	10.2
Does heartburn wake you from sleep?	123	41.7	50	16.9	53	18.0	34	11.5	17	5.8	18	6.1
Do you have difficulty swallowing?	156	52.9	35	11.9	53	18.0	29	9.8	12	4.1	10	3.4
Do you have pain while swallowing?	172	58.3	41	13.9	45	15.3	16	5.4	13	4.4	8	2.7
How bad is the regurgitation?	77	26.1	48	16.3	68	23.1	40	13.6	28	9.5	34	11.5

Table 3 presents the prevalence of regurgitation symptoms among the patients who underwent SG in Al-Qassim region, Saudi Arabia. The results show that 26.1% of the patients reported no symptoms of regurgitation, while 11.5% reported an inability to perform daily activities due to regurgitation. Regurgitation when lying down was reported by 31.5% of the patients, and regurgitation when standing up was reported by 49.5% of the patients. In terms of regurgitation after meals, 21.7% of the patients reported noticeable symptoms, but not bothersome, while 12.5% reported bothersome symptoms daily. Changes in diet due to regurgitation were reported by 37.6% of the patients. 46.1% of the patients reported that regurgitation woke them from sleep.

**Table 3:** The prevalence of regurgitation among the patients

	No symptoms		Noticeable , but not bothersome		Noticeable, bothersome but not every day		Bothersome daily		Bothersome and affects daily activities		Inability to perform daily activities	
	Count	Row, N%	Count	Row, N%	Count	Row, N%	Count	Row, N%	Count	Row, N%	Count	Row, N%
How bad is the regurgitation?	77	26.1	48	16.3	68	23.1	40	13.6	28	9.5	34	11.5
Regurgitation when lying down	93	31.5	47	15.9	64	21.7	40	13.6	19	6.4	32	10.8
regurgitation when standing up?	146	49.5	50	16.9	49	16.6	35	11.9	7	2.4	8	2.7
Regurgitation after meals?	88	29.8	54	18.3	64	21.7	37	12.5	20	6.8	32	10.8
Does regurgitation change your diet	111	37.6	52	17.6	52	17.6	37	12.5	14	4.7	29	9.8
Does regurgitation wake you from sleep?	136	46.1	37	12.5	61	20.7	26	8.8	13	4.4	22	7.5

**Figure 1:**
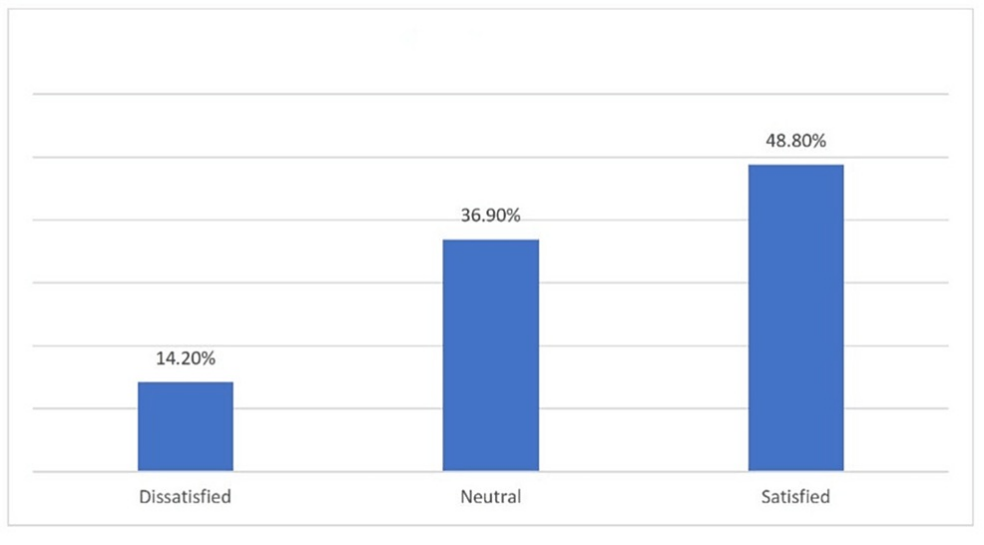
How satisfied are you with your current health ?

Figure 1 shows the distribution of patient satisfaction with their current health status. The study included 290 patients who underwent SG in Al-Qassim region, Saudi Arabia. The results show that 48.8% of the patients reported being satisfied with their current health status, while 36.9% reported feeling neutral. Only 14.2% of the patients reported being dissatisfied with their current health status.

**Figure 2:**
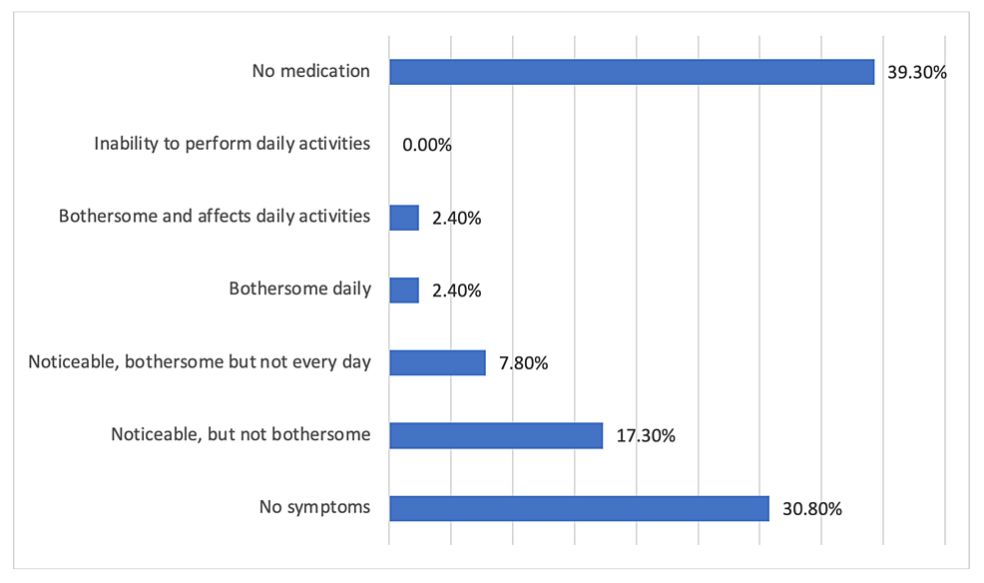
If you take medication, to what extent your symptoms improve?

Moreover, as shown in Figure 2, 30.8% of the participants reported that symptoms disappeared upon using of anti-reflex medications, while 17.3% reported having noticeable but not bothersome symptoms and 7.8% reported having noticeable, bothersome but not everyday symptoms.

Table 4 presents the association between demographic factors and the presence and severity of heartburn symptoms among patients who underwent SG in Al-Qassim region, Saudi Arabia. The results show that gender was not significantly associated with the presence and severity of heartburn symptoms (p-value=0.079). However, age was found to be significantly associated with heartburn symptoms (p-value=0.000*). Patients aged 25-35 years reported the highest prevalence of heartburn symptoms, followed by patients aged 36-45 years and patients aged >45 years. Smoker status was not found to be significantly associated with heartburn symptoms (p-value=0.605). The date of bariatric surgery was also found to be significantly associated with heartburn symptoms (p-value=0.001*). Patients who underwent bariatric surgery less than one year ago had the highest prevalence of heartburn symptoms, followed by patients who underwent surgery one to two years ago, three years ago, four years ago, five years ago, and more than five years ago. In addition, it was found that the use of the anti-reflux medications is significantly associated with how bad the heartburn (p=0.0001), where the more severe symptoms, the more prevalent of using reflex medications. Moreover, there is a significant relation between the severity of symptoms and whether the symptoms disappeared after medications (p=0.0001).

**Table 4:** The association between demographic factors and presence and severity of heartburn

		How bad is the heartburn ?																						
		No symptoms			Noticeable , but not bothersome					Noticeable, bothersome but not every day				Bothersome daily			Bothersome and affects daily activities				Inability to perform daily activities			
		Count		Row, N%	Count		Row, N%			Count		Row, N%		Count		Row, N%	Count			Row, N%	Count			Row, N%
Gender	Male	48		28.4	39		23.1			40		23.7		21		12.4	11			6.5	10			5.9
	Female	17		13.5	38		30.2			32		25.4		21		16.7	9			7.1	9			7.1
	P-value	0.079																						
Age	Less than 25	30		40.0	18		24.0			19		25.3		7		9.3	1			1.3	0			0.0
	25-35	22		20.6	25		23.4			26		24.3		16		15.0	10			9.3	8			7.5
	36-45	7		10.6	16		24.2			17		25.8		9		13.6	8			12.1	9			13.6
	> 45	6		12.8	18		38.3			10		21.3		10		21.3	1			2.1	2			4.3
	P-value	0.000*																						
Are you smoker?	No	43		20.7	59		28.4			49		23.6		32		15.4	13			6.3	12			5.8
	Yes	22		25.3	18		20.7			23		26.4		10		11.5	7			8.0	7			8.0
	P-value	0.605																						
The date of the bariatric surgery	Less than one year	27		44.3	14		23.0			10		16.4		5		8.2	3			4.9	2			3.3
	1-2 years	12		16.7	26		36.1			20		27.8		9		12.5	3			4.2	2			2.8
	3 years	4		11.4	13		37.1			8		22.9		7		20.0	2			5.7	1			2.9
	4 years	2		7.7	3		11.5			10		38.5		6		23.1	2			7.7	3			11.5
	5 years	2		10.5	3		15.8			4		21.1		3		15.8	4			21.1	3			15.8
	> 5 years	18		22.0	18		22.0			20		24.4		12		14.6	6			7.3	8			9.8
	P-value	0.001*																						
Do you take any reflex medication?	No	43	32.6			48		36.4	23		17.4		12		9.1			2	1.5			4	3.0	
	Yes	22	13.5			29		17.8	49		30.1		30		18.4			18	11.0			15	9.2	
	P-value	0.000*																						
if you take medication, to what extent your symptoms improve?	No symptoms	22	24.2			19		20.9	19		20.9		17		18.7			10	11.0			4	4.4	
	Noticeable, but not bothersome	2	3.9			13		25.5	20		39.2		7		13.7			4	7.8			5	9.8	
	Noticeable, bothersome but not every day	0	0.0			2		8.7	11		47.8		7		30.4			0	0.0			3	13.0	
	Bothersome daily	1	14.3			1		14.3	0		0.0		1		14.3			3	42.9			1	14.3	
	Bothersome and affects daily activities	1	14.3			1		14.3	2		28.6		0		0.0			1	14.3			2	28.6	
	Inability to perform daily activities	0	0.0			0		0.0	0		0.0		0		0.0			0	0.0			0	0.0	
	No medication	39	33.6			41		35.3	20		17.2		10		8.6			2	1.7			4	3.4	
	P-value	0.000*																						

## Discussion

SG is a common bariatric surgery treatment that has been proven beneficial for achieving significant weight loss and reducing obesity-related comorbidities [8-11]. However, SG can also result in a number of postoperative problems, such as heartburn and regurgitation [12,13]. These symptoms can profoundly impact patients' quality of life and may necessitate further treatment. Consequently, it is crucial to evaluate the occurrence and severity of heartburn and regurgitation symptoms in patients who have had SG. The objective of this study was to determine the prevalence and severity of heartburn and regurgitation symptoms in patients who had undergone SG in the Al-Qassim region of Saudi Arabia. The purpose of the study was also to examine the relationship between demographic variables and the presence and severity of these symptoms.

Heartburn and regurgitation were prevalent complaints among patients who underwent SG, according to the findings of the study. In the current study, 73.9% of patients experienced symptoms of regurgitation, while 11.5% of patients reported an inability to perform everyday activities. 78% of the patients reported experiencing heartburn symptoms, and 6.4% reported being unable to do everyday tasks. Consistent with earlier reports of a high prevalence of heartburn and regurgitation symptoms after SG, these results indicate a high incidence of heartburn and regurgitation [3,14-18].

A considerable majority of patients experienced moderate to severe heartburn and regurgitation symptoms that interfered with their everyday activities. For instance, 9.5% of patients reported that regurgitation symptoms were bothersome and interfered with their daily activities, while 6.5% reported that heartburn symptoms were bothersome and interfered with their daily activities. These results indicate that heartburn and regurgitation symptoms can have a considerable impact on the quality of life of SG patients [3,13,19].

Additionally, the relationship between demographic characteristics and the presence and severity of heartburn and regurgitation symptoms was explored. Age and the date of bariatric surgery were important factors linked with the presence and severity of these symptoms, according to the findings. Patients between the ages of 25 and 35 reported the highest prevalence of heartburn symptoms, followed by those between the ages of 36 and 45 and those older than 45. This conclusion is consistent with prior research indicating that younger individuals who undergo SG have an increased likelihood of experiencing heartburn symptoms [20-22].

Also shown to be substantially linked with heartburn and regurgitation symptoms was the date of bariatric surgery. Patients who had bariatric surgery within the past year had the highest prevalence of these symptoms, followed by those who had surgery between one and two years ago, three years ago, four years ago, five years ago, and more than five years ago. This finding is consistent with prior research indicating a higher incidence of heartburn and regurgitation symptoms in the early postoperative period [23-25].

The study found no correlation between gender or smoking status and the presence and severity of acid reflux and regurgitation symptoms. Some prior researchers have found a higher prevalence of these symptoms among female patients and smokers, which contradicts our finding [26]. Other studies, however, have found no significant association between gender or smoking status and the severity of these symptoms [27,28].

The findings of this study have significant clinical implications for the treatment of heartburn and regurgitation symptoms in patients after SG. Due to the high prevalence and severity of these symptoms, routine evaluation and management of heartburn and regurgitation symptoms should be an integral element of postoperative care [29]. Younger patients and those who undergo surgery in the early postoperative period may be at a greater risk for developing heartburn and regurgitation symptoms and may require more active measures to manage these symptoms.

The current study had some limitations including the cross-sectional design of the study which makes it difficult to demonstrate a causal relationship between demographic characteristics and the presence and severity of heartburn and regurgitation symptoms. In addition, additional potential risk factors for these symptoms, such as preoperative comorbidities and medication use, were not investigated.

## Conclusions

In conclusion, individuals who have SG frequently experience heartburn and regurgitation symptoms, which can have a substantial influence on their quality of life. The presence and severity of these symptoms are significantly influenced by age and the timing of bariatric surgery. Routine evaluation and treatment of heartburn and regurgitation symptoms should be an integral component of postoperative care. There is a need for additional studies to discover other potential risk factors for these symptoms and to create effective treatments for them.
